# Mutation analysis of *VSX1* and *SOD1* in Iranian patients with keratoconus

**Published:** 2011-11-30

**Authors:** Samira Saee-Rad, Hassan Hashemi, Mohammad Miraftab, Mohammad Reza Noori-Daloii, Morteza Hashemzadeh Chaleshtori, Reza Raoofian, Fatemeh Jafari, Wayne Greene, Ghasem Fakhraie, Farhad Rezvan, Mansour Heidari

**Affiliations:** 1 Department of Medical Genetics, Tehran University of Medical Sciences, Tehran, Iran; 2 Farabi Eye Hospital, Eye Research Center, Tehran University of Medical Sciences, Tehran, Iran; 3 Noor Ophthalmology Research Center, Noor Eye Hospital, Tehran, Iran; 4 Cellular and Molecular Research Center, Shahrekord University of Medical Sciences, Shahrekord, Iran; 5 Biomedical Science, School of Veterinary and Biomedical Sciences, Murdoch University, South Street, Murdoch, Western Australia

## Abstract

**Purpose:**

To evaluate mutations in the visual system homeobox gene 1 (*VSX1*) and superoxide dismutase 1 (*SOD1*) genes with keratoconus (KTCN), direct sequencing was performed in an Iranian population.

**Methods:**

One hundred and twelve autosomal dominant KTCN patients and fifty-two unaffected individuals from twenty-six Iranian families, as well as one hundred healthy people as controls were enrolled. Genomic DNA was extracted from whole blood sample. Then to study the possible linkage between KTCN and six known loci linkage analysis was performed using 12 short tandem repeat (STR) markers. Also, the entire coding region and intron-exon boundaries of *VSX1* and *SOD1* were amplified by the PCR technique in each proband. Subsequently, PCR products were subjected to direct sequencing. Co-segregation analysis of the identified mutation was conducted in the family members. An Amplification Refractory Mutation System PCR (ARMS-PCR) was additionally employed for detection of the identified mutation in healthy controls.

**Results:**

Linkage analysis of aforementioned loci did not detect evidence for linkage to KTCN. Direct PCR sequencing revealed two single nucleotide polymorphisms (SNPs; g.1502T>G and g.9683C>T), as well as two missense mutations that have been previously reported (R166W and H244R) in *VSX1.* We also found three undescribed SNPs (g.4886G>A, g.4990C>G, and g.9061T>A) in *SOD1*. The R166W and H244R mutations were co-segregated in affected family members but not in those that were unaffected. Moreover, the ARMS-PCR strategy did not detect the identified mutations in controls.

**Conclusions:**

Our data suggest a significant association between KTCN patients and *VSX1* genetic alterations (p.R166W and p.H244R). Although our findings support *VSX1* as a plausible candidate gene responsible for keratoconus, other chromosomal loci and genes could be involved in KTCN development. Taken together, our results suggest that p.R166W and p.H244R could have possible pathogenic influences on KTCN.

## Introduction

Keratoconus (KTCN; OMIM 148300) is a genetically and clinically heterogeneous disease affecting the cornea that causes distortion and reduced vision. KTCN is the most common indication for corneal transplantation in developed countries [1,2]. The estimated incidence is between 1 in 500 and 1 in 2,000 individuals in the general population and the prevalence is estimated to be 54.5 per 100,000 [1]. It occurs in both genders and all ethnicities [3]. Studies suggest that the prevalence and incidence rates are higher in Asians compared to Caucasians [4,5]. A study undertaken in the Midlands area of the UK (UK), a prevalence of 4:1, and an incidence of 4.4:1 was reported in Asians compared to Caucasians while another UK study conducted in Yorkshire, found that the incidence was 7.5 times higher in Asians compared to Caucasians [6].

Several lines of evidence support the importance of genetic components in the pathogenesis of KTCN [6-8]. It has been shown that the prevalence of KTCN in first-degree relatives is significantly higher than the general population [4,5]. Approximately 6% to 23.5% of cases had familial transmission [9] which were inherited in either an X-linked or autosomal recessive or dominant trait. About 90% of pedigrees with familial KTCN exhibit an autosomal dominant inheritance with reduced penetrance, the age of onset in teenage years and variable clinical expression [1,4,6]. In addition, it has been well documented that KTCN is associated with syndromic conditions such as connective tissue disorders (osteogenesis imperfecta, Gapo syndrome, and some subtypes of Ehlers-Danlos syndrome) [1], pigmentary retinopathy, Marfan’s syndrome, Noonan’s syndrome, Apert’s syndrome, Leber congenital amaurosis, and Down syndrome [2,4,10].

KTCN appears to be a genetically heterogeneous disorder as several chromosomal regions and genes are suggested to be involved in the molecular etiology of KTCN. To date, many different chromosomal loci including 20p11-q11 (KTCN1; OMIM 148300) [11], 16q22.3-q23.1 (KTCN2; OMIM 608932) [12], 3p14-q13 (KTCN3; OMIM 608586) [13], 2p24 (KTCN4; OMIM 609271) [14], 15q22.32–24 [15,16], and 5q14.3-q21 [17], as well as genes such as *VSX1* (visual system homeobox) and *SOD1* (superoxide dismutase) have been implicated in KTCN pathogenesis [3,4,18-23]. In addition to genetic factors, environmental factors as well as genetic-environmental interactions could play critical roles in KTCN [9].

In this study, we conducted mutation detection of the entire *VSX1* and *SOD1* codon sequences in 112 patients from twenty-six Iranian families. Our results support the possible pathogenic function of *VSX1* genetic variants in probands of two families with KTCN. To confirm the association of the identified VSX1 variants, we performed co-segregation analysis in affected family members and in controls. To our knowledge, this is the first report of an association between KTCN and *VSX1* genetic alterations in Iranian patients.

## Methods

### Ophthalmological examination

This study was approved by the local Institutional Review Board (IRB) and informed consent was obtained from all affected individuals. Keratoconus was diagnosed based on the following criteria: (1) distortion of the corneal surface; (2) progressive visual acuity reduction; (3) progressive stromal thinning within central cornea using a comprehensive ophthalmic examination including, visual acuity measurement, corneal imaging including topography, and Pentacam imaging (Noor Eye Hospital, Tehran, Iran).

### Molecular genetic studies

Five milliliters of peripheral blood was collected in test tubes containing 0.5 M EDTA. DNA was extracted using a QIAamp DNA mini kit (Qiagen, Hilden, Germany). Genotyping of all family members using twelve short tandem repeat (STR) markers (Table 1) from different chromosomal regions was conducted as previously reported [12-14,19,24]. Briefly, PCR amplification was typically performed in 25 μl PCR reactions, 1 U *Taq* DNA polymerase, 10 pmole/µl of each primer, (information on the sequences of markers, PCR primers and data on related polymorphic fragments was obtained from the UCSC genome browser,), 200 μM of dNTPs, 0.67 μl of 50 mM MgCl_2_, 60 ng DNA and 2.5 μl of 10× PCR buffer). The PCR conditions included an initial denaturation step for 3 min at 95 °C, 30 s at 95 °C, 45 s at 64 °C with a 1 °C decrease every second cycle down to 55 °C, then 55 °C for 14 cycles, 1 min at 72 °C for extension, and finally 10 min at 72 °C. PCR-amplified products in genotyping were separated on 12% polyacrylamide gels and the bands detected using silver staining as previously described [25]. Briefly, the gel was fixed in a solution consists of  10% acetic acid and stained with a solution containing silver nitrate and formaldehyde for 30 min. Subsequently, the gel was rinsed and developed in an ice-cold alkaline sodium carbonate solution containing formaldehyde and sodium thiosulphate.

**Table 1:** List of STR markers used in this study.

**Marker**	**Position**	**CM**	**Chr**
D16S2624	56051189–56051331 (bp)	87.62 (cM)	16q22.3-q23.1
D16S3090	61948845–61949107 (bp)	92.10 (cM)	16q22.3-q23.1
D3S1312	62406410–62406632 (bp)	82.24 (cM)	3p14.2
D3S3683	110636567–110636738 (bp)	127.89 (cM)	3q13.2
D5S2499	87363565–87363812 (bp)	102.62 (cM)	5q
D5S495	94814389–94814609 (bp)	108.07 (cM)	5q
D2S305	19099786–19100000 (bp)	38.87(cM)	2p24.2
D2S2373	20842807–20842972 (bp)	42.65(cM)	2p25-p22
D20S119	40357700–40357811 (bp)	61.77 (cM)	20q11.2-q13.2
D20S838	41348847–41348967 (bp)	64.88 (cM)	20q11.2-q13.1
Cyp11a1	51426178–51426904 (bp)	60.31 (cM)	15q23-q24
D15S211	57951161–57951385 (bp)	75.85 (cM)	15q24

Mutation screening was performed for the complete coding regions of *VSX1* and *SOD1* using specific primers (Table 2) under the same PCR conditions as above. PCR products were directly sequenced (Gene, Fanavaran, Iran). Sequence data searches were performed in non-redundant nucleic and protein databases BLAST. Also, in this study, we used different softwares such as Chromas, HaploPainter V.024 Beta, easyLinkage PLUS v5.05, and Cyrillic 2.1.

**Table 2:** List of *VSX1* and *SOD1* primers and predicted PCR product sizes (bp) used in this study.

**Primer**	**Sequence (5'→3')**	**PCR product size (bp)**	**Primer location**
***VSX1* Primer sequences**			
V1F	5′-GCAGCCCAATCCTATAAAGC-3′	687	1–20
V1R	5′-GATTACCGGACGTGGAGA-3′		469–486
V2F	5′-AAGTCCTCTTCTTCTTTCTGTGCCATC-3′	800	2610–2637
V2R	5′-AAGGGACTGCTGATTGGCTCACTG-3′		3386- 3409
V3F	5′-ATCATGCTCGGGAGAGAAGA-3′	487	4178–4197
V3R	5′-AAAATGAGGCAACCATCCAG-3′		4639–4660
V4F	5′-CCAATGCCAATCACTGTGTC3′	306	5366–5385
V4R	5′-CCCAGAGTCCTGCCAACTTA-3′		5652–5672
V5F	5′-AGGAAGTGAAGATAAGTTGGCAG-3′	470	5640–5662
V5R	5′-TAAAGTGCCATTAAGGAACCG-3′		6110–6090
V6F	5′-AACGGTTCCTTAATGGCACTT-3′	301	6088–6109
V6R	5′-TTGAAATATCCAAGGCCAAGTT-3′		6367–6388
V7F	5′-ATCATAGTGAAGACTCCATACAGACA-3′	424	6312–6337
V7R	5′-AGCCCTCACAATGAGCAGTT-3′		6964–6984
V8F	5′-GAGGCAGCATCTCAGGACTT-3′	534	9834–9854
V8R	5′-AGGTGTGAGGTACAGGTCCAA-3′		9321–9340
V9F	5′-GCTCAGGTAGCATTGTTCTGC-3′	610	10272–10292
V9R	5′-TGATGGAAGGAGAGGAGAAGG-3′		10861–10881
**ARMS primers**			
VSM	5′-AGTCTGGCAGCGAGATGTAGC-3′	236	4392–4411
VSWT	5′-ACTGCATCCCGCTGCCAGACT-3′		43911–4411
VSF	5′-GGATCATGCTCGGGAGAGAAGA-3′		4176–4197
***SOD1* primer sequences**			
S1F	5′-CTCCACATTTCGGGGTTCT-3′	450	4850–4868
S1R	5′-ACCCGCTCCTAGCAAAGGT-3′		5281–5292
S2F	5′-CCATCTCCCTTTTGAGGACA-3′	426	8965–8985
S2R	5′-CGACAGAGCAAGACCCTTTC-3′		9371–9390
S3F	5′-TGATGCAGGTCAGCACTTTC-3′	344	11717–11736
S3R	5′-AAAAGCATTCCAGCATTTGG-3′		12041–12060
S4F	5′-CCATCTTTCTTCCCAGAGCA-3′	386	12810–12840
S4R	5′-GAAACCGCGACTAACAATCAA-3		12454–12473
S5F	5′-TTTGGGTATTGTTGGGAGGA-3′	675	13780–13799
S5R	5′-TCTGTTCCACTGAAGCTGTTT-3′		14334–14355

### Amplification refractory mutation system PCR (ARMS-PCR)

The ARMS-PCR method was applied for the screening of identified mutations in *VSX1* in unaffected and control individuals. Primer sequences are shown in Table 2. Mismatches were included to maximize discrimination of the wild-type and mutant alleles. Amplifications were performed as outlined above. All samples under investigation were analyzed simultaneously alongside positive and negative controls for the R166W and H244R mutations. To determine the genotype results, amplification products were resolved on 2% agarose gels stained with ethidium bromide.

## Results

Probands from 26 Iranian families were identified with KTCN. All affected and controls were born after a normal term pregnancy. Retinoscopy, corneal topography, and Pentacam examinations of probands’ first-degree relatives discriminated KTCN from normal individuals. Affected cases were clinically examined and showed no signs and symptoms of any syndromic indication. KTCN was excluded in normal controls by retinoscopy, corneal topography, and Pentacam examination.

### Linkage analysis results

To investigate the association of six known (20p11-q11, 16q22.3-q23, 3p14-q13, 2p24, 15q22.32–24, and 5q14.3-q21) genetic loci with KTCN, genomic DNA was isolated from 112 affected 52 unaffected family members. First, PCR primers were used to amplify polymorphic markers (Table 1) on chromosome 20p11-q11, 16q22.3-q23, 3p14-q13, 2p24, 15q22.32–24, and 5q14.3-q21 known to be linked to KTCN. Then, PCR products were separated by PAGE. Haplotype analysis could not define linkage between known loci with KTCN in the studied families (as shown in Appendix 1).

### Mutation analysis of *VSX1*

**Figure 1:**
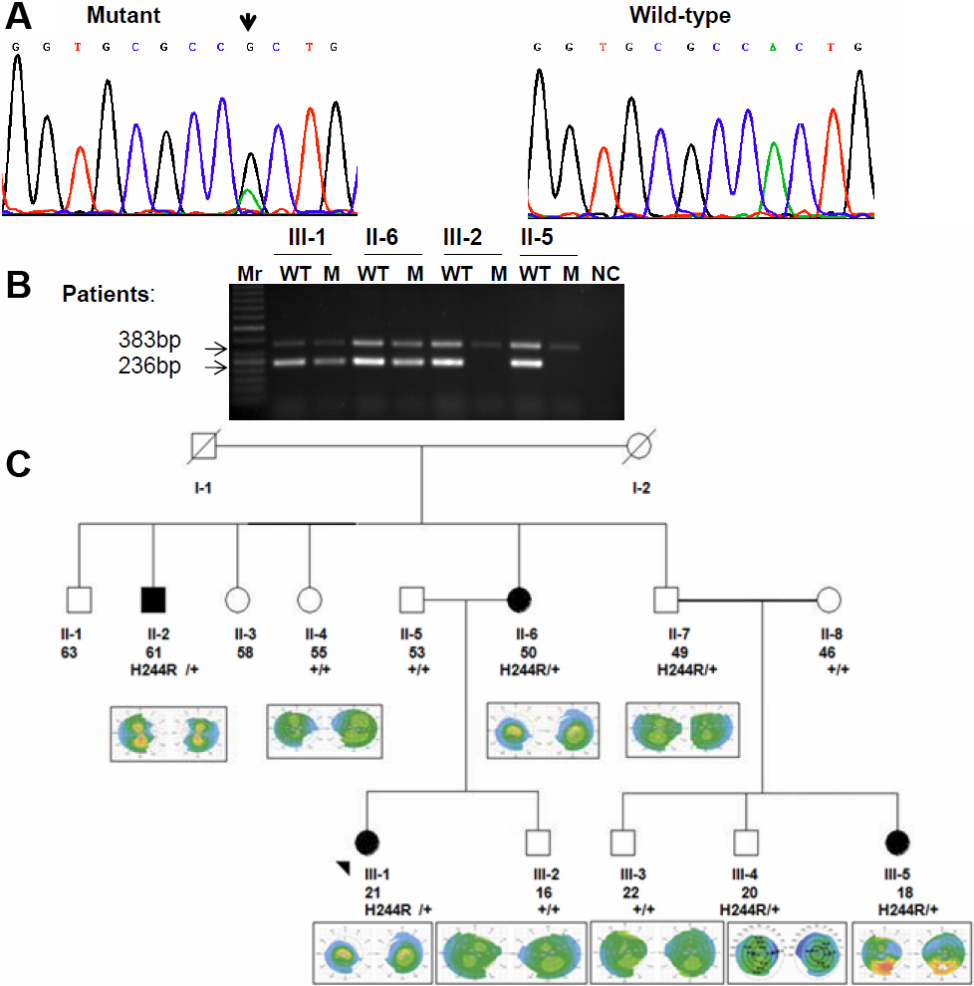
Pedigree analysis and molecular study of Family 1. **A**: DNA sequencing revealed heterozygous missense mutation in the codon 244 *VSX1* in which A→G (arrow indicates the position of nucleotide substitution). **B**: Amplification refractory mutation system (ARMS) for H244R *VSX1* genotyping showing the co-segregation of the H244R *VSX1* mutation among family members including two KTCN patients (III:1 and II:6) as well as in two individuals without KTCN clinical features (III:2 and II:5). PCR products of the internal control primer pair (383 bp), PCR product of the wild-type (WT) and mutant primer pairs (236 bp) are indicated. M, 50-bp ladder is present. **C**: The pedigree of Family 1 show four affected patients (arrow indicates the proband) and segregation of p.H244R through the family. Each individual was reported by age (in years), genotype and topography images. Filled symbols represent KTCN patient and open symbols reveal individuals without clinical KTCN.

**Figure 2:**
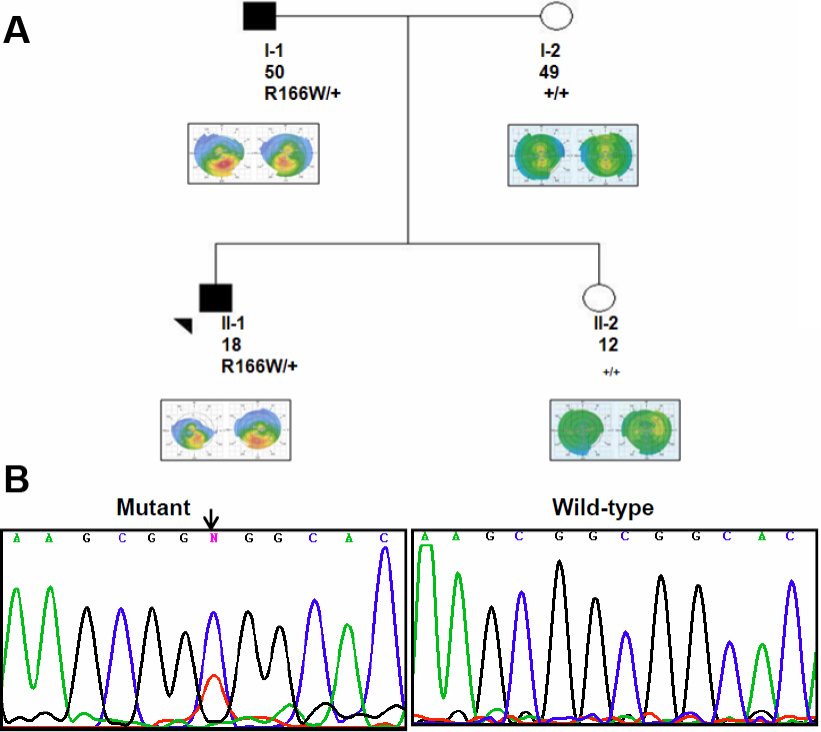
Pedigree analysis and molecular study of Family 2. **A**: DNA sequencing chromatogram from heterozygous mutant of proband (II:1) showed missense mutation in codon 166 in which Arg was replaced by Trp (R166W C>T; arrow indicates the position of nucleotide substitution). **B**: The Pedigree of Family 2 indicates two affected patients (arrow indicates the proband) as well as the segregation of p.R166W in the family. The each family's member was presented by age (in years), genotype and topography images. Filled symbols show KTCN patient while open symbols represent persons without clinical KTCN.

Direct PCR sequencing using forward and reverse primers (Table2) was conducted to evaluate genetic alterations in the coding sequence and exon/intron boundaries of *VSX1*. We found two single nucleotide polymorphisms (SNPs) (g.1502T>G and g.9683C>T) and two non-synonymous mutations (H244R and R166W) in *VSX1* (Figure 1 and Figure 2).

In family 1, the H244R mutation was identified in a 21-year-old female. The proband had three affected relatives (Figure 1A). To test the pathologic function of this genetic mutation, co-segregation of H244R (Figure 1B) in other affected family members were performed using PCR sequencing. We also screened the H244 R *VSX1* mutation in 100 controls by ARMS-PCR (Figure 1C). Direct sequencing and ARMS-PCR results confirmed that only affected patients carried p.H244R in the heterozygous state, while Figure 1B (right) indicates the wild-type (WT) variant of *VSX1* gene at codon position 244.

In family 2, the R166W mutation was detected in an 18-year-old male (Figure 2A). Figure 2B indicates R166W in II-1 (proband) and his father (I-1). Although the proband and his father presented a variable expressivity of KTCN, the R166W co-segregated among KTCN, but not in unaffected his mother.

### Mutation analysis of *SOD1*

We additionally evaluated a possible association of KTCN with *SOD1* genetic alterations. The full-length *SOD1* coding sequence was screened by direct PCR sequencing. In spite of the fact that three novel SNPs (g.4886G>A, g.4990C>G, and g.9061T>A) were identified in non-coding sequences, however, sequencing the coding region did not reveal a sequence variant segregating with disease in any of the families described. The SNPs did not seem to influence the activity of SOD1 protein.

## Discussion

Various genome-wide linkage analyses and mutation detection studies have reported that six loci and two genes (*SOD1* and *VSX1*) are thought to be associated with KTCN in different ethnic groups [6]. In this study, we performed linkage analysis for six known chromosomal loci as well as a mutation detection screen in *VSX1* and *SOD1* in 112 affected, 52 unaffected family members and 100 normal individuals as controls in a group of Iranian patients with keratoconus. All studied families displayed an autosomal dominant pattern of KTCN with variable expressivity. According to literature KTCN is a complex disorder with multiplfactorial etiology. It has been well documented that genetic and environmental factors are associated with this disease. Different studies have shown that about 90 percent of pedigrees with familial KTCN display an autosomal dominant inheritance with incomplete penetrance [11,16]. Our pedigree analysis showed a reduced penetrance (about 58%) among Iranian families with KTCN.

Genotyping results using linkage analysis failed to detect any significant correlations between known loci and KTCN. Direct PCR sequencing did not show important genetic changes in *SOD*1, although three novel SNPs were found in the probands of three families with KTCN.

For *VSX1*, we observed two previously undescribed SNPs (g.1502T>G and g.9683C>T) in addition to two missense mutations that had been previously reported (R166W and H244R) [11].

The *VSX1* homeobox gene encodes a homeodomain transcription factor that may regulate expression of the cone opsin genes early in normal development [26].Despite the fact that *VSX1* remains the only major genetic element to be identified in KTCN pathogenesis, there has been debate in the literature in regard to its pathobiological function in KTCN. So far, several VSX1 protein coding changes (p.L17P, p.D144E, p.L159M, p.G160D, p.R166W, p.H244R, and p.P247R) have been reported as being potentially pathogenic in KTCN [3,27,28]. Valleix et al. [29] reported that H244R *VSX1* is associated with selective cone ON bipolar cell dysfunction and macular degeneration in a posterior polymorphous corneal dystrophy (PPCD) family [29]. However, different studies in several ethnic groups [3,30,31] did not detect any mutations in *VSX1* [21,32,33].

To evaluate the possible function of p.H244R genetic alteration we undertook co-segregation analysis and mutation detection in the subject families and 100 unaffected individuals, respectively. The mutation was not observed in 100 unaffected people who were examined by corneal topography followed by Pentacam evaluation. Therefore, for first time, our results support the notion that p.H244R could represent a pathogenic change in KTCN, although it has been previously identified [11]. Héon et al. [11] showed that the *VSX1* mutation p.H244R co-segregated with the disease in two family members. However, they also observed this genetic change in two controls (n=277). For that reason, they suggested that this change may or may not be disease-causing [11]. These results could be due to several factors, such as the mode of inheritance, gene-gene interaction, gene-environment interaction, and genetic background. About 90% of pedigrees with familial KTCN are predominately transmitted in an autosomal-dominant fashion with an incomplete penetrance and variable expressivity [9]. A comparison of the findings obtained in our study and from other studies raises a critical question: what could be the biologic significance of the observation of *VSX1* H244R alteration in KTCN? With respect to this event, there are several possibilities that may account for the presence of the *VSX1* H244R change observed in our family including:

1. random appearance in the affected members without any biologic significance,
2. the H244R mutation may not directly cause the disease but its pathogenic functions could be due to gene-gene interactions, gene-environment interactions, or genetic background, or
3. the presence of this change in the unaffected individual suggests that this could be a causative mutation with incomplete penetrance.

Our results suggest that the H244R *VSX1* change may be a pathogenic variant with incomplete penetrance.

The R166W *VSX1* mutation was also initially found in an isolated case of keratoconus with visual impairment for whom a corneal graft was required in adulthood [11]. We observed this mutation in the proband of a family with two affected patients with KTCN. Our results showed that R166W was co-segregated in two affected family members, but not in unaffected individuals and controls. The R166W alters the highly conserved third amino acid of the DNA binding homeodomain (HD). Dorval et al. [34] suggested that this mutation causes keratoconus in humans by impairing VSX1 DNA binding [34].

Three SNPs were identified in non-coding sequences of the *SOD1* gene. This gene is located on chromosome 21 and functions to destroy free superoxide radicals in the body [22]. The association of *SOD1* mutation with KTCN was originally reported by Udar et al. [22]. *SOD1* mutations were screened in 15 unrelated individuals, each with a family history of KTCN. Results from this study determined an IVS2+50del7 change within intron 2 in two families. Also, they observed that this 7-base deletion segregated with the KTCN subjects in a studied pedigree [22]. However, an independent study failed to define any association between *SOD1* mutations and KTCN [34]. Stabuc-Silih and colleagues [33] studied the association of KTCN with *VSX1* and *SOD1* gene mutations in 113 Slovenian patients with sporadic and familial KTCN by direct sequencing. They found no causative disease mutation in the *SOD1* gene but a significant association was detected between a *VSX1* polymorphism (627+23G>A) and KTCN [33]. Our findings are in agreement with published data suggesting that other genetic and non-genetic factors are involved the pathogenesis of KTCN.

We conclude that the R166W and H244R *VSX1* variants might play critical roles in the pathogenesis of KTCN. To test the potential pathogenic relevance of these variants we performed co-segregation analysis in all affected and unaffected family members. To rule out the incomplete penetrance of autosomal dominant KTCN in unaffected family members, we conducted precise clinical examinations of unaffected individuals; a crucial step in an association study involving a molecular genetic analysis of an autosomal dominant disorder. An accurate diagnosis and exclusion of keratoconus in patients and controls (n=100), respectively, was made using corneal topography and Pentacam evaluation. Our findings suggest that the R166W and H2446R mutations might be involved in KTCN pathogenesis.

## Appendix 1. Twenty six pedigrees of KTCN.

To access the data, click or select the words “Appendix 1.” This will initiate the download of a compressed (pdf) archive that contains the file.
